# A novel hybrid deep learning pipeline for precise segmentation of skin cancer lesions

**DOI:** 10.3389/fmed.2026.1915861

**Published:** 2026-08-20

**Authors:** Xian-Hong Wang, Muhammad Saeed, Naeem Ahmed, Wen-Ke Di, Xin-Ying Ji, Ping-Ping Xu, Umair Ali Khan Saddozai

**Affiliations:** 1 Henan Provincial Research Center of Engineering Technology for Nuclear Protein Medical Detection, Department of Internal Medicine and Pediatrics, Medical School, Zhengzhou Health College, Mazhai, Erqi, Zhengzhou, Henan, China; 2 School of Software, Nanjing University of Information Science and Technology, Nanjing, China; 3 Department of Dermatology, Shandong Medical College, Lanshan, Linyi, Shandong, China; 4 Henan International Joint Laboratory for Nuclear Protein Regulation, Department of Nuclear Medicine, The First Affiliated Hospital, Henan University College of Medicine, Kaifeng, Henan, China; 5 Henan Provincial Research Center of Engineering Technology for Nuclear Protein Medical Detection, Department of Microbiology and Immunology, Zhengzhou Health College, Zhengzhou, Henan, China; 6 Henan Provincial Research Center of Engineering and Technology for Medical Detection of Nuclear Protein, School of Medicine, Zhengzhou Health College, Mazhai, Erqi, Zhengzhou, Henan, China; 7 Medical College, Institute of Translational Medicine, Yangzhou University, Yangzhou, Jiangsu, China

**Keywords:** deep learning, hybrid CNN-ViT architecture, medical image analysis, skin lesion segmentation, vision transformers

## Abstract

**Introduction:**

Skin cancer is among the most prevalent malignancies worldwide, and accurate segmentation of skin lesions plays a vital role in its early diagnosis and treatment. Traditional deep learning models face challenges in balancing local feature extraction with global contextual understanding. This study aims to develop a novel, robust, and efficient hybrid model that combines lightweight CNNs and ViTs to enhance the accuracy and reliability of automated skin lesion segmentation for use in diverse clinical settings.

**Methods:**

The proposed framework is divided into several stages. The first stage is data preparation, which ensures consistency across datasets by standardizing input images. The second stage employs a lightweight CNN module to extract important local features from the input images. These features are then passed to a ViT module to capture long-range dependencies and global context. Finally, a segmentation head processes the combined features to produce the final lesion mask. Three publicly available datasets (ISIC 2020, PH2, HAM10000) were used for training, testing, and generalization evaluation.

**Results:**

The proposed hybrid architecture achieved an accuracy of 95.89%, a Dice coefficient of 94.29%, and a Jaccard index of 96.91% using the SGD optimizer on the ISIC 2020 dataset, outperforming several baseline and state-of-the-art methods. Cross-dataset evaluation on PH2 and HAM10000 yielded accuracies of 94.61% and 95.68%, respectively.

**Discussion:**

The proposed hybrid model presents a promising solution for accurate and efficient skin lesion segmentation, contributing to enhanced clinical decision-making in dermatological diagnostics.

## 1 Introduction

Millions of people worldwide suffer from skin diseases that not only diminish quality of life but also lead to increased discomfort and rising healthcare costs. In the United States alone, skin lesions affect millions, contributing to a spectrum of chronic conditions that often coexist with other dermatological issues. (1). These lesions can result from genetic factors and immune system irregularities that compromise the integrity of the epidermal barrier, leading to dryness, inflammation, and a heightened risk of infection (2). Accurate and efficient skin lesion segmentation is essential for early diagnosis and effective treatment, which supports timely clinical intervention and improved patient outcomes (3).

Traditional techniques for skin lesion detection are time-consuming and subjective. These techniques often depend on interobserver variability (4). Medical imaging has gone through various stages and advancements in the past few decades. After the introduction of Convolutional neural networks (CNN), most of the techniques use them as a key component for extracting complex spatial features. CNN performs well in extracting spatial features from dermoscopic images necessary for skin lesion detection and segmentation (5). However, traditional CNN methods often face challenges in capturing global contextual relationships and long-range dependencies. Contextual relationships and long-range dependencies are essential for accurately detecting the abnormal part and identifying the boundaries of skin lesions (6).

After the CNN Vision Transformers (ViTs) were introduced for better medical image analysis. The ViTs uses attention mechanisms that effectively capture global relationships in images. In this way, ViTs overcomes the limitations of traditional CNNs and achieves better results in medical image analysis (7). After studying various techniques based on these advancements and their limitations in the literature, we proposed a novel hybrid model for skin lesion detection and segmentation. The proposed model uses a lightweight CNN module with a Vision Transformer module. The CNN component is used to extract local features from dermoscopic images, while the vision transformer module is used for long-term contextual relationships. This combination ensures that both the global structure and intricate details are maintained throughout the segmentation process (8).

We divide the proposed framework into various stages. The first stage is data preparation, which ensures consistency across various datasets by standardizing input images. The second phase develops the lightweight CNN module for extracting important local features from the input images. These features are then sent to the ViT module to capture long-range dependencies and global context to enhance the representations. Finally, a segmentation head is used to process these combined features to create the final lesion mask. We used three well-known publicly available datasets to train and test the proposed system. Moreover, separate datasets are also used to test the generalizability of the model after training and testing. The key contributions of our work are summarized as follows:

- We proposed a computationally efficient hybrid CNN–Vision Transformer architecture that extracts discriminative local lesion features using lightweight convolutional blocks while modeling global contextual dependencies through transformer-based self-attention.
- We present a sequential CNN-to-ViT feature integration strategy in which hierarchical convolutional representations are transformed into global attention embeddings before decoder reconstruction, enabling improved lesion boundary localization without introducing excessive architectural complexity.
- We present an end-to-end segmentation framework consisting of standardized preprocessing, lightweight feature extraction, transformer-based contextual modeling, and decoder-based mask reconstruction for precise skin lesion segmentation.
- We used comprehensive cross-dataset validation using ISIC 2020 for model development and PH2 and HAM10000 for external evaluation, demonstrating robustness and strong generalization compared with existing CNN-based and hybrid segmentation approaches.

Unlike existing hybrid CNN–Transformer segmentation models, the proposed framework emphasizes an efficient sequential CNN-to-ViT feature learning strategy that balances local feature extraction with global contextual modeling while maintaining low computational complexity. In addition, its effectiveness is validated through cross-dataset evaluation on ISIC 2020, PH2, and HAM10000, demonstrating strong robustness and generalization.

The proposed framework focuses on enhancing the identification of early skin lesions by addressing both the extraction of local features and the integration of global contextual knowledge. The rest of the article is divided into 5 Sections. Section contains the literature review, while Section 3 presents the process and architecture of the proposed framework. Section 4 discusses the Results and discussion part of the study, while Section 5 concludes the article.

## 2 Literature review

Skin lesion segmentation is a key process for the diagnosis of various diseases. In recent years, deep learning techniques and the evolution of computer vision have changed medical image analysis processing. Medical image segmentation is one of the key areas where we can use computer vision and intelligent techniques. These techniques reduce healthcare costs and prognosis times for dermatological conditions. Moreover, they are very crucial for early diagnosis, treatment planning, and improved patient outcomes (9).

As discussed, earlier CNNs were used very frequently before the introduction of advanced techniques. Their use is due to their flexibility and reliable results. Early CNN models combine pooling operations and sequential convolution to capture both low-level and high-level semantic features. With a deeper network, CNNs can encode complex representations and can detect complex patterns that are crucial for accurate lesion detection A CNN-based segmentation technique was presented by Yanagisawa et al. (9) for medical images segmentation. Their model draws boundaries around lesions and produces a segmented image dataset that can be used to train more complex classification models like Inception-V3. Several specialized architectures for image segmentation have also improved performance. A notable example is the U-Net design, which has gained popularity in medical image segmentation due to its effective skip connections and encoder-decoder structure.

Araujo et al. (11) presented a CNN-based U-Net model that achieves a handsome segmentation accuracy on dermoscopic images while using minimal computational resources. In order to take advantage of both spatial and sequential data properties, researchers have also looked into hybrid models that mix CNNs with Long Short-Term Memory (LSTM) networks. Sadik et al. established a framework that combines CNNs with LSTM models, employing advanced preprocessing methods like data augmentation and enhancement to boost segmentation and disease recognition. In a similar vein, Balaha et al. introduced a meta-heuristic optimization strategy, the Sparrow Search Algorithm, alongside various U-Net models and pre-trained CNNS, highlighting the advantages of merging different model architectures to enhance segmentation accuracy. While traditional U-Net architectures have gained widespread use, their inability to capture intricate spatial details and manage diverse lesion forms points to the need for architectural improvements. Recent progress emphasizes refining feature extraction, maintaining spatial information, and optimizing decoder structures to elevate segmentation accuracy.

Similarly, beyond deep CNNs, traditional machine learning techniques have also been applied to skin lesion segmentation. Nisar et al. (12) used supervised learning methods including Support Vector Machines, Naïve Bayesian Classifiers, and K-Nearest Neighbors on images acquired from both clinical settings and online sources. Although these methods can be effective, their performance is often limited by the need for extensive manual feature engineering and preprocessing. Despite all these advancements, Automated segmentation still faces challenges due to lesion variability in size, shape, texture, and boundary ambiguity.

The encoder path in segmentation models is pivotal for extracting hierarchical features. While shallow U-Net encoders struggle with complex lesion patterns, deeper convolutional networks like ResNet by He et al. (13) and DenseNet by Huang et al. (14) have shown promise in capturing high-level features. For instance, ResUNet by Zhang et al. (15). Integrated residual blocks into U-Net, improving gradient flow and feature reuse. Similarly, Jha et al. (16) demonstrated that pretrained VGG16 encoders enhanced melanoma segmentation on the ISIC dataset by leveraging transfer learning. However, deeper architecture often incurs computational costs, limiting its clinical applicability. Hybrid approaches, such as MobileNetV3-based encoders by Howard et al. (17) balance depth and efficiency but sacrifice sensitivity to subtle lesion textures.

Skip connections in U-Net bridge semantic gaps between encoder and decoder, but standard implementations lose fine details for small or irregular lesions. Recent studies propose augmented skip connections to mitigate this. Attention U-Net by Oktay et al. (18) introduced gating mechanisms to filter irrelevant features, while Zhou et al. (19) employed nested dense connections to enrich gradient propagation. Despite these advances, boundary blurring persists in lesions with low contrast against the surrounding skin. Dual skip connections and multi-scale feature fusion by Li et al. (10) improved boundary delineation in dermoscopic images but increased model complexity.

The decoder's role in reconstructing spatial maps from encoder features is often underexplored. Traditional U-Net decoders underutilize high-dimensional features, leading to suboptimal lesion recovery. Recent works like DoubleU-Net by Jha et al. (16) stacked two decoders to refine outputs, while TransUNet Chen et al. (20) combined transformers with U-Net decoders for global context modeling. However, these methods neglect channel capacity alignment between encoder and decoder.

Models trained on single datasets often fail to generalize due to dataset-specific biases, as demonstrated in large-scale studies of dermatological AI systems (21) PH2 by Mendonça et al. (22) with its high-resolution histopathological images, and ISIC (23), with diverse dermoscopic samples, represent complementary challenges. While Al-Masni et al. (24) achieved strong ISIC performance using cascaded CNNs, their model faltered on PH2′s finer structures. Similarly, hybrid framework by Gessert et al. (25) prioritized lesion detection over boundary precision (26). More recent advances have further expanded skin lesion segmentation research, including diffusion-based bridge models, spectral generalized transformers, SAM-powered multi-scale adaptation frameworks, boundary-guided lightweight networks, uncertainty estimation methods, and state-space modeling with convolutional perception (27–33).

## 3 Methodology

**Figure 1:**
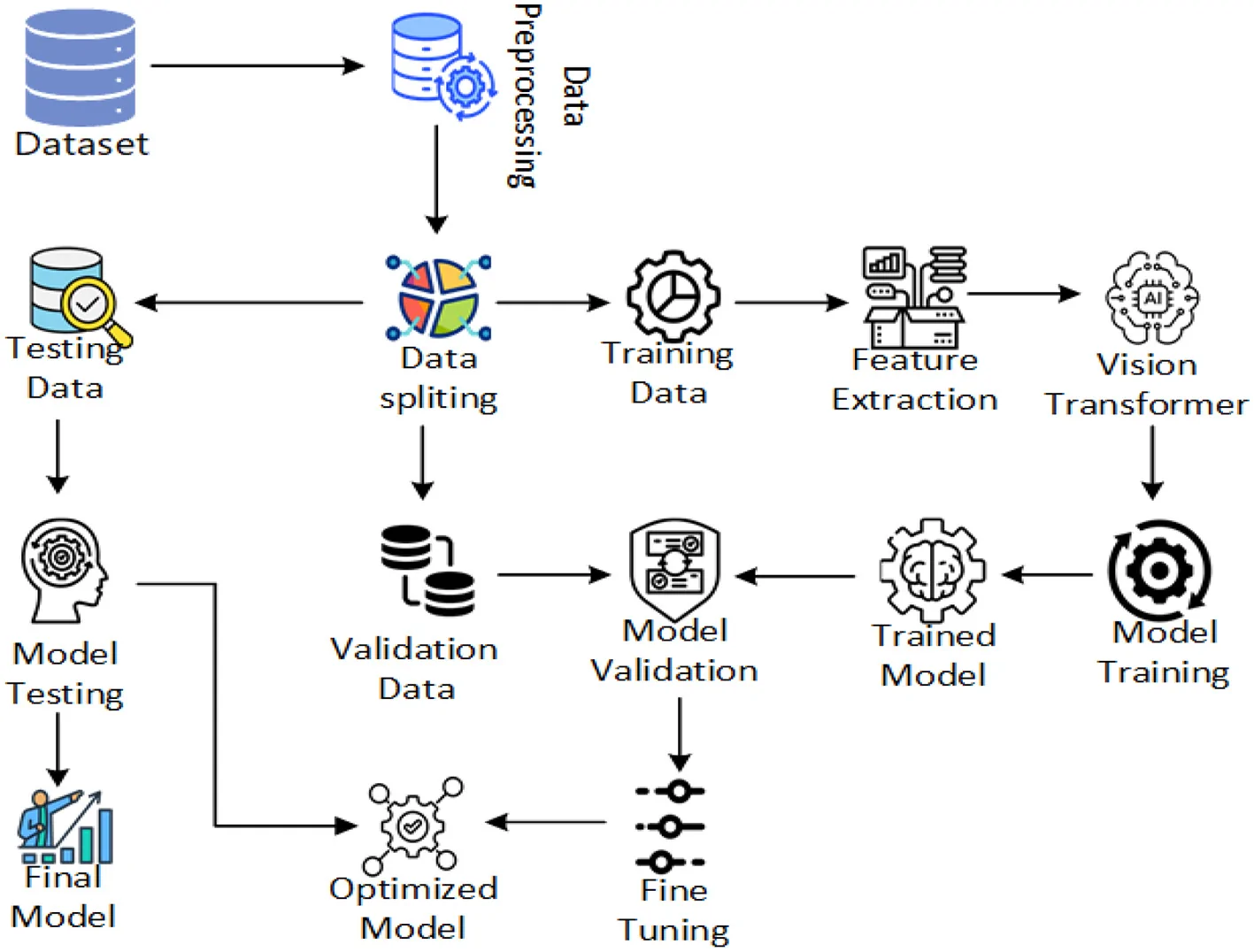
Overview of the proposed model.

A hybrid segmentation model that integrates lightweight CNN layers with Vision Transformer modules to accurately detect and segment skin lesions from images was developed. The primary objective is to classify the presence of skin disease and delineate the affected regions. Figure 1 illustrates the proposed process for skin lesion segmentation.

The framework starts with the collection of image datasets, and then we pre-processed the images so that they could be processed by the next step. After preprocessing, the data is split into training and testing subsets. The preprocessing step also includes a Gaussian smoothing technique to reduce noise and enhance lesion clarity.

After the preparation of data, we applied the lightweight CNN model for local feature extraction. The output of the CNN module is then given to the ViT module that focuses on extracting focus on global contextual relationships between features of the input data. The proposed model was trained iteratively until it reached an optimal accuracy level. After the optimization, we used the model for the segmentation of RGB images. We also used the separate datasets for testing the generalizability of the proposed model. This comprehensive process enables us to develop a reliable and robust segmentation framework. The ISIC 2020 dataset that is known as a standard for skin lesion detection and segmentation, was used in the experiments. Moreover, the PH2 and HAM10000 datasets were utilized to test the model generalizability and efficiency on unseen data. Table 1 provides an overview of these datasets.

**Table 1:** Overview of the datasets.

Datasets	Number of images
ISIC 2020	33,126
PH2	200
HAM10000	10,015

**Figure 2:**
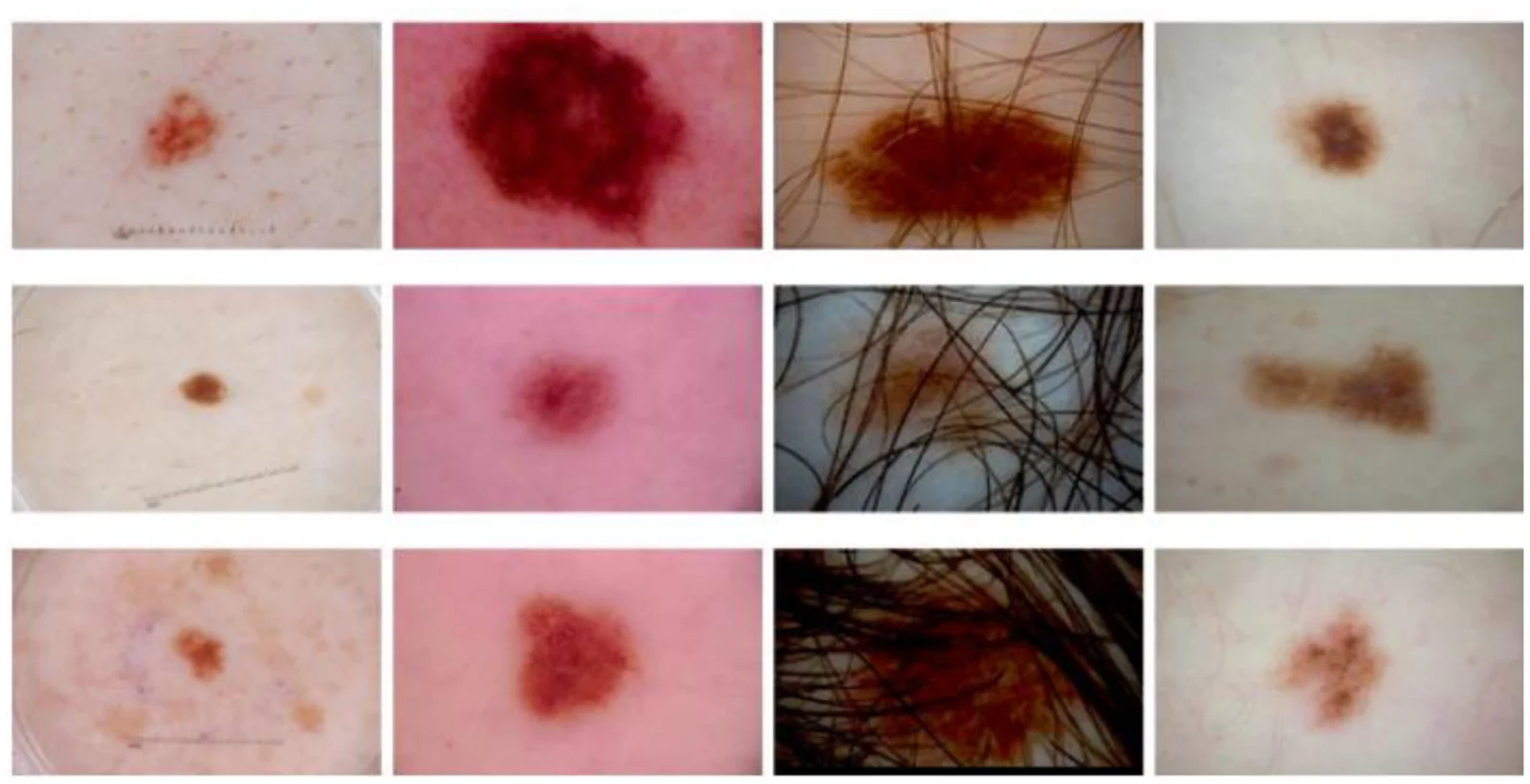
Sample of ISIC dataset.

The dataset contains 33,126 dermoscopic training images of unique benign and malignant skin lesions from over 2,000 patients. Each image is associated with one of these individuals using a unique patient identifier. All malignant diagnoses have been confirmed via histopathology, and benign diagnoses have been confirmed using either expert agreement, longitudinal follow-up, or histopathology. Figure 2 illustrates to some sample images from ISIC 2020 dataset.

**Figure 3:**
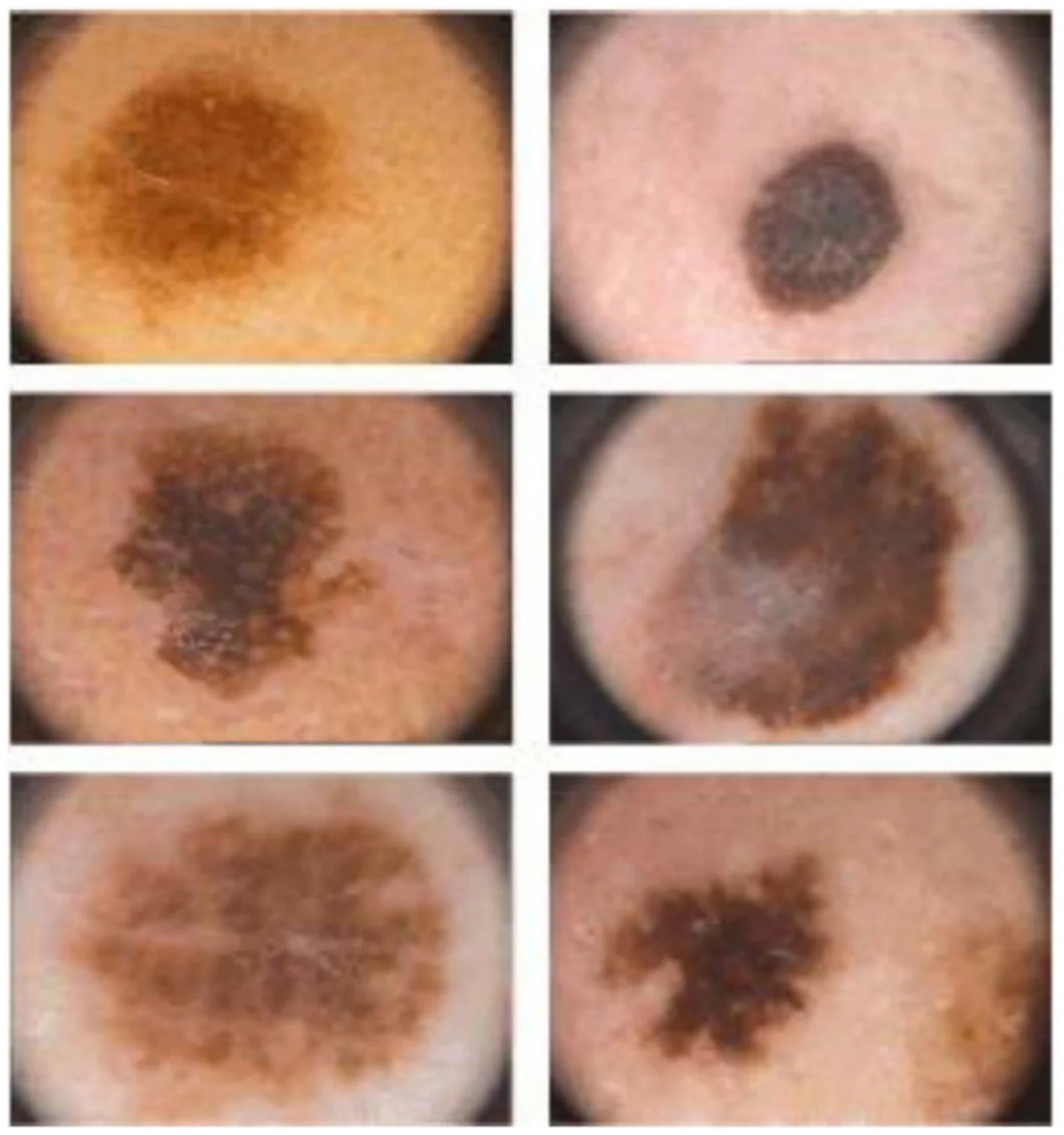
Some images from PH2 dataset.

Figure 3 presents few images from the PH2 dataset. The PH2 dataset was developed for research and benchmarking purposes to facilitate comparative studies of segmentation and classification algorithms for dermatoscopic images. PH^2^ is a database of dermatoscopic images obtained from the Dermatology Service at Pedro Hispano Hospital in Matosinhos, Portugal. This dataset contains 200 dermatoscopic images along with corresponding annotation images, all stored in PNG format.

**Figure 4:**
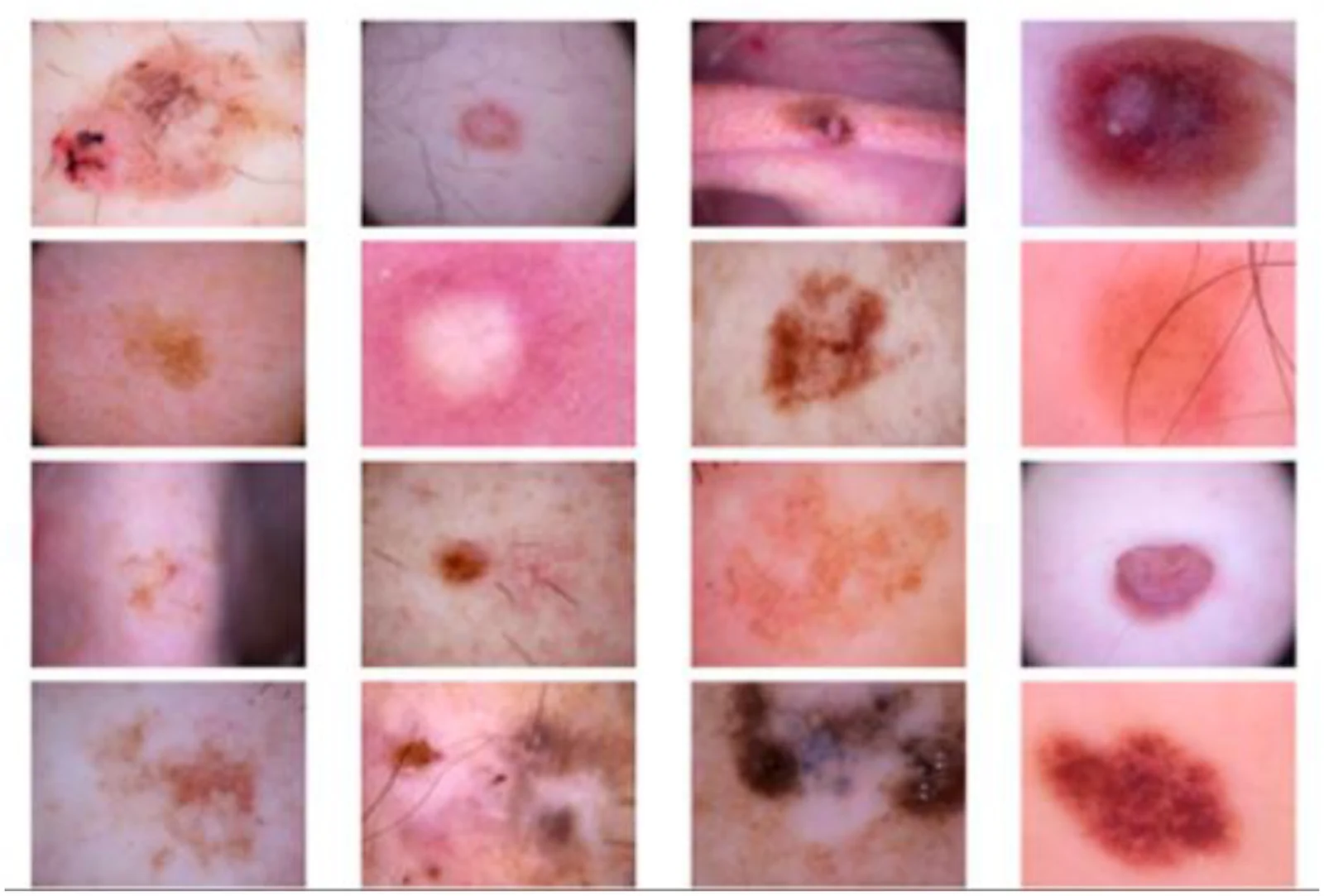
Sample of HAM10000 dataset.

Some sample images from Ham1000 dataset are shown in Figure 4. This dataset is used for testing the gernalization of the proposed model. This dataset consists of 10,015 images with 10,013 labeled objects belonging to 7 different classes including melanocytic nevi, melanoma, benign keratosis-like lesions, and other: basal cell carcinoma, actinic keratoses, vascular lesions, and dermatofibroma.

### 3.1 Experimental setup

To ensure experimental reproducibility, all experiments were conducted using fixed training settings. The proposed hybrid CNN–ViT model was trained for 100 epochs with a mini-batch size of 8 and initial learning rate of 5 × 10^−5^. Optimization of the model was carried out with the AdamW and Stochastic Gradient Descent (SGD) optimizers. AdamW was chosen due to its adaptive learning feature and good weight regularization; and SGD was chosen for comparison. Binary Cross Entropy with Logits Loss (BCEWithLogitsLoss) was used to optimize the segmentation model. All the input dermoscopic images were resized to 192 × 256, normalized and smoothed using a Gaussian filter prior to training. The datasets were split into training, validation and testing sets of 70%, 10% and 20% respectively. The proposed framework is implemented in PyTorch framework and trained on NVIDIA RTX 4090D GPU.

We used various evaluation metrics, including the Dice coefficient, Jaccard index, accuracy, precision, and recall, to evaluate the segmentation accuracy and performance.

### 3.2 Overview of proposed hybrid model

The proposed hybrid model integrates lightweight CNN layers for local feature extraction with Vision Transformer modules to capture global context, followed by a segmentation head that refines lesion boundaries. The following procedure (Algorithm 1) outlines the step-by-step process for skin lesion segmentation using this architecture.

Algorithm 1 Hybrid CNN–ViT Segmentation Framework for Skin Lesions. 
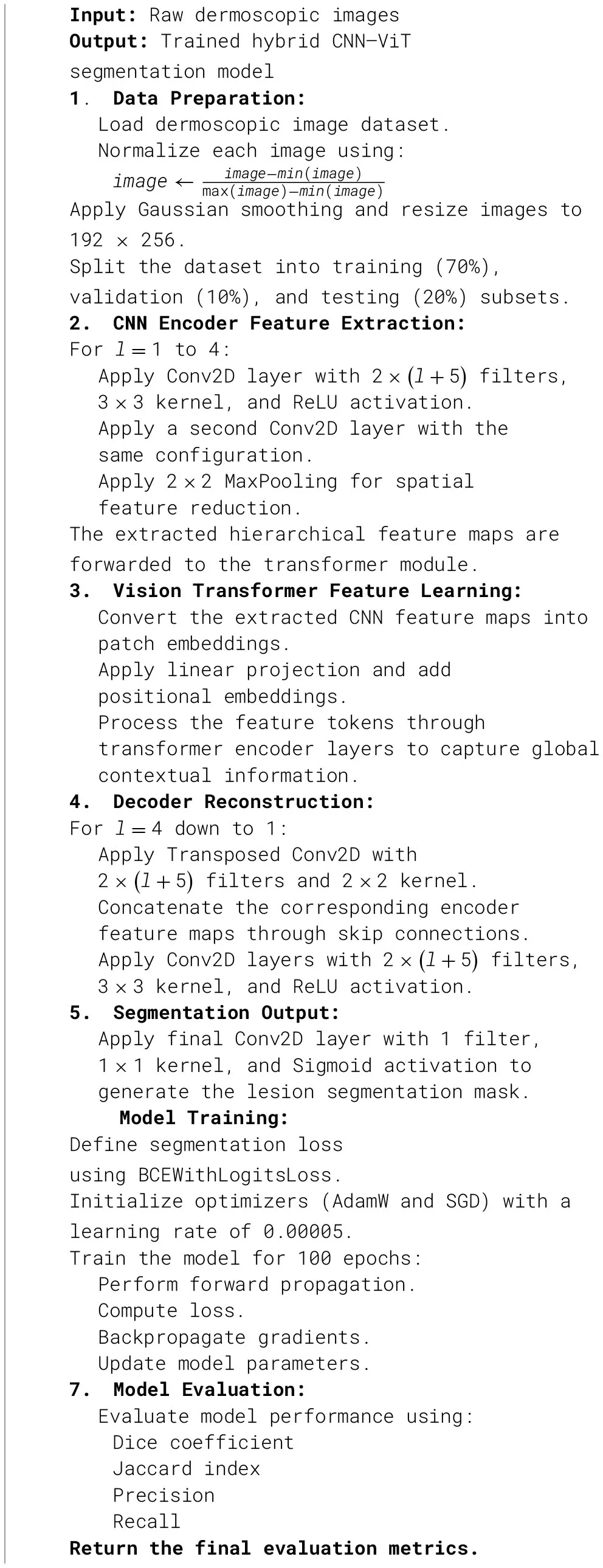

The above algorithm describes the process of extracting both local features through CNN layers and global context learning using Vision Transformer layers.

The proposed hybrid model combines lightweight CNN for initial feature extraction with Vision Transformer (ViT) layers for global attention-based representation learning. The overall framework of ViT consists of two main steps. The first one is an encoder for feature extraction, and the second is a decoder for precise segmentation.

After effectively extracting local features using lightweight convolutional layers, the encoder down-samples spatial dimensions via max-pooling operations. This method creates hierarchical representations by removing less important regions from the image and capturing fine-grained features and abstracted information. Vision Transformer (ViT) layers are then used to offer attention-based learning over the entire image to capture global dependencies.

The ViT encoder enhances how the model understands the connections between distant parts of an image. This step enables more accurate lesion segmentation by highlighting important areas in the entire context.

The decoder performs precise upsampling to create pixel-level segmentation maps. This upsampling uses transposed convolutions to transform the compressed feature representations into detailed segmentation outputs. The hybrid architecture effectively captures intricate details and expansive background information, enabling accurate localization of lesion boundaries. This setup supports pixel-wise segmentation, with the model's design ensuring that the input and output are sequentially aligned for precise characterization. We employ a single-stage model that correctly classifies lesions and produces accurate boundaries, thereby generating a complete segmentation pipeline.

#### 3.2.1 Data preparation

The preprocessing step is crucial for standardizing and enhancing the input images before they are fed into the model. We used several transformations, including resizing, normalization, and noise reduction using Gaussian smoothing. The input image *I* ∈ ℝ^*H*×*W*×*C*^ is resized to a fixed size (*H*′, *W*′) where *H*′ and *W*′ are the height and width of images to ensure uniformity. The resizing process is represented in Equation 1.


$$\begin{array}{l} I^{\prime }=Resize\left(I,\left(H^{\prime },W^{\prime }\right)\right) \end{array}$$ (1)

After this step, we applied normalization on the input images. The normalization process can be defined by Equation 2.


$$I^{'}norm=\frac{I^{'}-\mu }{\sigma }$$ (2)

Here, μ and σ represent the mean and standard deviation of the dataset, respectively. This standardization ensures that the pixel values are centered around zero, with a consistent scale. Gaussian smoothing is applied to reduce high-frequency noise and smooth out the image. This operation uses a Gaussian filter to blur the image to suppress noise while preserving edges. The process can be mathematically represented as in Equation 3.


$$\begin{array}{l} G\left(x,y\right)=\frac{1}{2\pi \sigma^{2}}\exp\left(-\;\frac{x^{2}+y^{2}}{2\sigma^{2}}\right) \end{array}$$ (3)

where σ s is the standard deviation of the Gaussian distribution for controlling the extent of the smoothing. The *k* is kernel filter, and the Gaussian filter is convolved with the image $I_{norm}^{'}$ as given by Equation 4.


$$I^{'}smooth=I^{'}norm\;*\;G(x,y)$$ (4)

where *is used to represent the convolution operation. The kernel G(x,y) is applied across the entire image which results in a smoothed version of the normalized image. where *is used to represent the convolution operation. The kernel G(x,y) is applied across the entire image, that results in a smoothed version of the normalized image.

#### 3.2.2 Vision transformer configuration

The ViT module is added post the light-weight CNN encoder module to capture long-range dependency from the extracted lesion features. CNN feature maps are flattened and projected sequentially and positional embeddings are added to retain the spatial information. The transformer encoder has a residual connection, multiple heads of self-attention blocks, FFN blocks, and layer norm for stable optimization. To make it reproducible, the ViT module has an embedding dimension of 256, 8 attention heads and 4 transformer encoder blocks. Self-attention layers, MLP layers with GELU activation, and a dropout rate of 0.1 to prevent overfitting are built into each block. These learned contextual representations are then fed to the decoder and we obtain the final pixel-level lesion segmentation mask using transposed convolutions and skip connections.

#### 3.2.3 Decoder for segmentation

The decoder takes the output of the ViT encoder and generates the pixel-wise segmentation mask. The decoder performs symmetric upsampling through transposed convolutions (also known as deconvolutions). The process of upsampling is given by:


$$\begin{array}{l} X_{upsampled}=TransConv\left(X_{vit},W_{deconv}\right)+b_{deconv} \end{array}$$ (5)

Where, *W*_*deconv*_ and *b*_*deconv*_ are the weights and biases of the transposed convolution operation, and *X*_*upsampled*_ is the upsampled feature map. Finally, the segmentation head performs a 1 × 1 convolution to map the output classes.


$$\begin{array}{l} S=Conv\left(X_{upsampled},W_{seg}\right)+b_{seg} \end{array}$$ (6)

Where, *S* ∈ ℝ^*H*^′ × *W*′ × 1 represents the segmentation output, which is a binary mask indicating the presence of skin lesions. Table 2 presents the summary of proposed model architecture.

**Table 2:** Summary of the proposed framework.

Layer type	Details	Output shape
**Input**	Raw dermoscopic image, resized and normalized	*H*×*W*×*C*
**Preprocessing**	Normalize, Gaussian smoothing, resize to 192 × 256	192 × 256 × *C*
**Encoder Block** (Layer 1)	Conv2D (10 filters, 3 × 3), ReLU, MaxPooling (2 × 2)	96 × 128 × 10
**Encoder Block** (Layer 2)	Conv2D (12 filters, 3 × 3), ReLU, MaxPooling (2 × 2)	48 × 64 × 12
**Encoder Block** (Layer 3)	Conv2D (14 filters, 3 × 3), ReLU, MaxPooling (2 × 2)	24 × 32 × 14
**Encoder Block** (Layer 4)	Conv2D (16 filters, 3 × 3), ReLU, MaxPooling (2 × 2)	12 × 16 × 16
**CNN to Patch Flattening**	Flatten features, project, add positional encoding	*N*×*D*
**Vision Transformer Encoder**	Self-attention layers	*N*×*D*
**Decoder Block** (Layer 4)	Transposed Conv2D (16 filters, 2 × 2), concatenate, Conv2D	12 × 16 × 16
**Decoder Block** (Layer 3)	Transposed Conv2D (14 filters, 2 × 2), concatenate, Conv2D	24 × 32 × 14
**Decoder Block** (Layer 2)	Transposed Conv2D (12 filters, 2 × 2), concatenate, Conv2D	48 × 64 × 12
**Decoder Block** (Layer 1)	Transposed Conv2D (10 filters, 2 × 2), concatenate, Conv2D	96 × 128 × 10
**Output Layer**	Conv2D (1 filter, 1 × 1), Sigmoid activation	192 × 256 × 1

## 4 Results and discussion

The experimental results highlight the effectiveness of the proposed Hybrid CNN-ViT Segmentation Model in accurately segmenting skin lesions. The evaluation was conducted on three benchmark datasets: ISIC 2020, HAM10000, and PH2. The ISIC 2020 dataset was used to train the proposed system. After successful training, we used two well-known datasets, i.e., PH2 and HAM10000, to assess the proposed system generalizability. Our aim was to make a generalizable system, so this process enabled us to perform a more thorough evaluation of the model's performance. The results of the proposed system show its applicability to diverse medical scenarios.

The proposed model was trained using 100 epochs by carefully analyzing the convergence and learning complex patterns from the data. During the training, we used two well-known optimizers, i.e., AdamW and Stochastic Gradient Descent (SGD). These optimizers were used to determine the optimizer's impact on the performance of the proposed model. We select the AdamW optimizer due to its adaptive learning rate, while SGD was used to analyze its effectiveness and good results in traditional optimization tasks.

Various standard metrics was used for the evaluation of proposed model after training phase. These metrics includes Accuracy, Dice Score, Jaccard Index (IoU), Precision, and Recall. These metrics provide valuable insights into the model's ability to segment skin images.

### 4.1 Comparison with state-of-the-art methods

In order to further validate the effectiveness of the proposed CNN–ViT framework, the proposed method is compared with the recent state-of-the-art skin lesion segmentation methods. Selected methods consist of traditional CNN based models, attention-based networks, and Transformer based architectures. The comparison is made based on standard segmentation measures. The summary of the results is presented in Table 3.

**Table 3:** Comparison of the proposed CNN–ViT framework with recent state-of-the-art skin lesion segmentation methods.

Method	Backbone	Dataset	Dice (%)	Jaccard/IoU (%)
U-Net	CNN	ISIC 2018	89.20	80.70
Attention U-Net	CNN + Attention	ISIC 2018	89.60	81.30
TransUNet	CNN + Transformer	ISIC 2018	88.50	79.90
Swin-UNet	Swin Transformer	ISIC 2018	88.50	79.90
CTH-Net	CNN + Transformer Hybrid	ISIC	93.40	81.90
SkinFormer	Texture Transformer	ISIC 2018	93.20	—
Att-Swin U-Net	Swin Transformer + Cross-Context Attention	ISIC	93.70	88.20
UCM-Net	MLP + CNN	ISIC 2017	—	80.71
UCM-Net	MLP + CNN	ISIC 2018	—	81.26
SkinAttn-Net	Multi-level Attention + ViT	ISIC 2017	92.60	86.30
SkinAttn-Net	Multi-level Attention + ViT	ISIC 2018	91.00	83.60
**Proposed CNN–ViT**	**CNN** **+** **Vision Transformer**	**ISIC 2020**	**94.59**	**94.43**

Bold values indicate the best performance among the compared methods.

**Figure 5:**
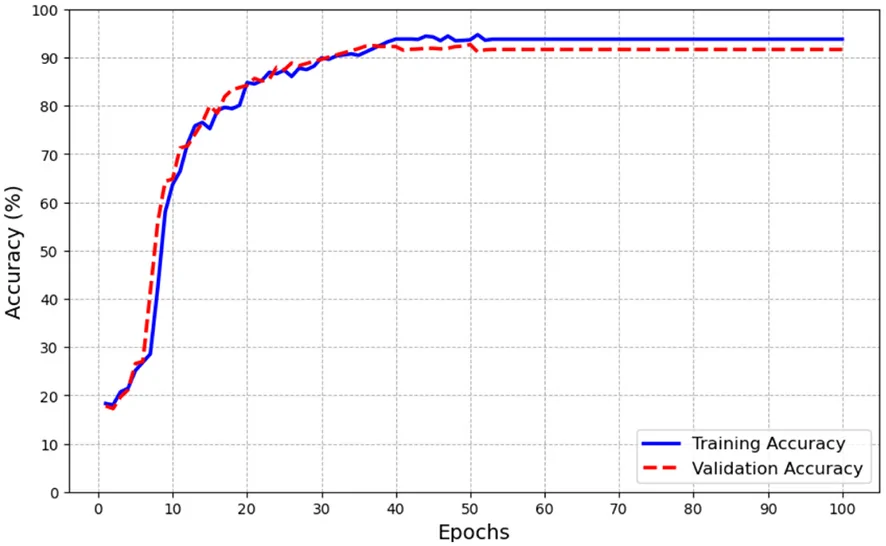
Accuracy of the proposed model using AdamW.

**Figure 6:**
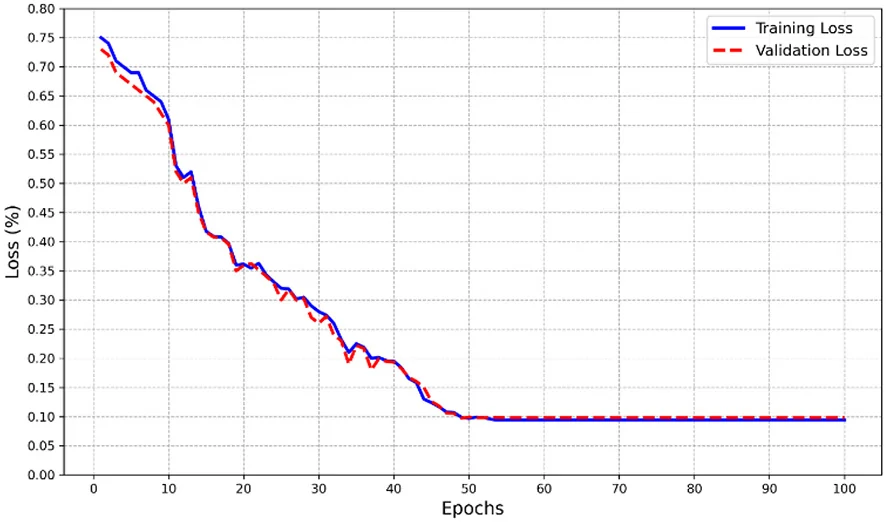
Model loss curve using AdamW.

As presented in Table 3, the proposed CNN–ViT model achieves competitive performance to the state-of-the-art CNN, attention, and Transformer-based segmentation approaches. The proposed approach achieves Dice score of 94.59% and Jaccard score of 94.43%, with enhanced lesion boundary localization and segmentation accuracy. This improvement is mainly due to the complementary strengths of CNNs in extracting fine-grained local features and Vision Transformers in capturing long-range dependencies. Figures 5, 6 illustrate the training and validation accuracy, along with loss values for the AdamW optimizer.

**Figure 7:**
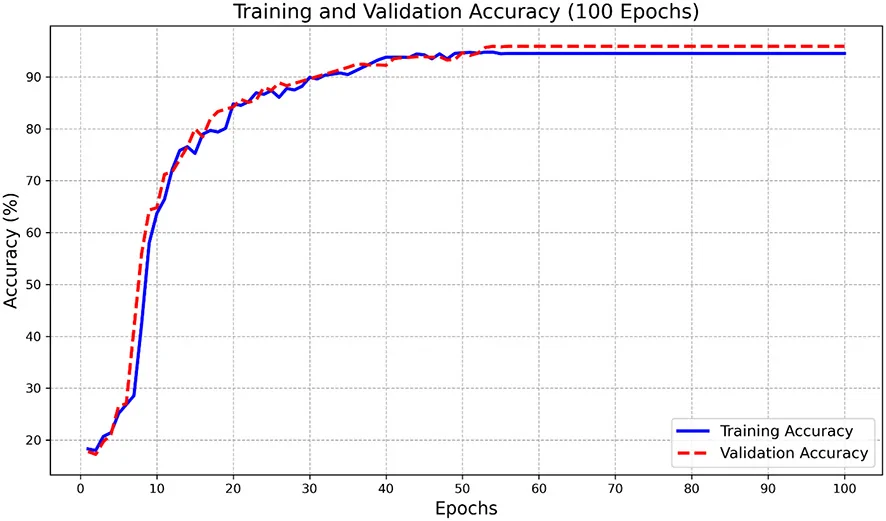
Accuracy of the proposed model using SGD.

**Figure 8:**
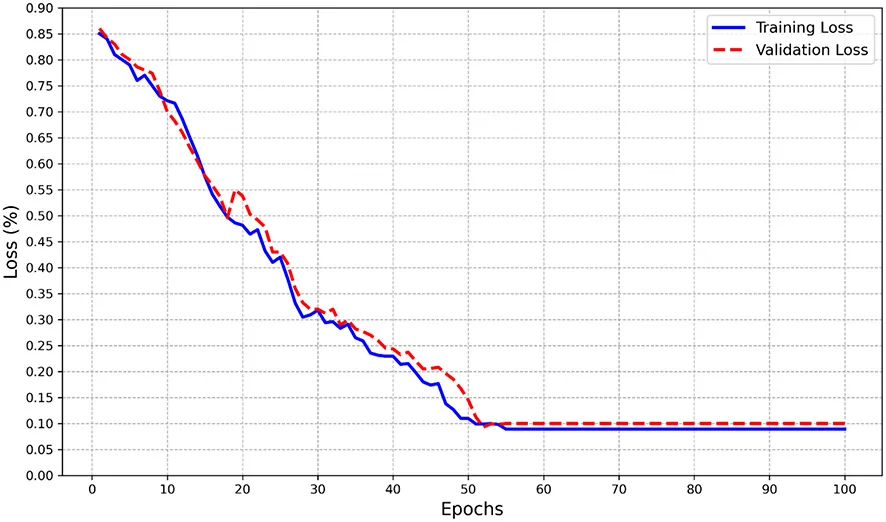
Model loss curve using SGD.

The training and validation losses of the proposed hybrid model are monitored to evaluate its effectiveness, as depicted in Figure 5. As demonstrated in Figures 5, 6, the model's accuracy improves progressively with the number of epochs, while the loss value decreases correspondingly. In these graphs, blue lines represent training metrics, and dotted red lines represent validation metrics. Moreover, the model is trained and validated using the SGD optimizer, with performance results illustrated in Figures 7, 8.

The accuracy curves for both training and validation phases show that the proposed model achieved 94.5% accuracy during training and 95.52% during validation with the SGD optimizer.

The training and validation loss curves highlight the model's steady improvement over successive epochs. The proposed model is further evaluated using various metrics, including the Dice coefficient, Jaccard index, precision, and recall, to comprehensively assess its segmentation performance. Table 4 presents the performance of the proposed model in the training phase with different optimizers.

**Table 4:** Performance of proposed model.

Metric	AdamW	SGD
Accuracy	93.76%	95.89%
Dice Score	94.59%	94.29%
Jaccard Score	94.43%	96.91%
Precision	94.31%	96.15%
Recall	95.13%	96.97%

The results from the training phase show that we can learn and adapt the intricate patterns of lesion images. The high rate of accuracy indicates that the model is good at the predictions of segmentations of pixels. The Dice Score is used to measure the spatial overlap between the predicted and actual masks. We also used the Jaccard Score to assess the similarity between the actual and predicted masks of the input image. The proposed model achieved 95.69% accuracy, which shows the model's capability to identify positive lesion cases accurately. The recall value of 96.97% confirms that it captures most actual positive cases. Together, these metrics highlight the strength and dependability of the proposed hybrid model. Table 5 summarizes the model's performance across testing and validation datasets.

**Table 5:** Comprehensive summary of model performance.

Metric	Validation (AdamW)	Validation (SGD)	Testing (AdamW)	Testing (SGD)
Accuracy	92.32%	96.21%	93.39%	95.56%
Dice Score	95.46%	94.42%	95.31%	94.87%
Jaccard Score	94.32%	96.89%	93.73%	95.99%
Precision	95.33%	96.88%	94.87%	96.61%
Recall	94.99%	97.23%	95.31%	96.98%

The Table 5 shows the effectiveness of proposed model on different dataset by using various evaluation metrics. The testing and validation stages evaluate the model's generalization ability using previously unseen data.

### 4.2 Model generalization

As previously stated, we utilized two distinct datasets to test the generalization capabilities of proposed framework. This experiment aimed to evaluate how well the model performs on entirely unfamiliar data, to confirm its adaptability and flexibility. The results of the proposed model on the PH2 dataset are presented in Table 6.

**Table 6:** Results of the proposed model on the PH2 dataset.

Metric	AdamW	SGD
Accuracy	93.88%	94.61%
Dice Score	95.89%	94.84%
Jaccard Score	93.83%	95.21%
Precision	93.21%	94.81%
Recall	92.17%	94.96%

In testing with the PH2 dataset, our model reached an impressive accuracy of 94.61%, demonstrating its strong ability to accurately predict skin lesion pixels. The performance of the segmentation is highlighted by Dice and Jaccard scores of 95.89% and 93.83%, respectively. Additionally, our precision and recall scores of 94.81% and 94.96% show that the model is effective at minimizing false positives while accurately identifying actual lesion cases. These results affirm the effectiveness and reliability of our system in detecting dermatological diseases. The results of for HAM10000 are given in Table 7.

**Table 7:** Results of the proposed model on HAM10000.

Metric	AdamW	SGD
Accuracy	94.38%	95.68%
Dice Score	95.99%	95.21%
Jaccard Score	94.37%	95.88%
Precision	92.86%	94.94%
Recall	93.49%	95.89%

The evaluation of the model using the HAM10000 dataset highlights its effectiveness and adaptability. With an impressive accuracy of 95.68%, the model shows its capability to segment skin lesion pixels accurately in diverse images. The Dice score of 95.99% and Jaccard score of 95.88% further emphasize its reliable detection abilities. In addition, the precision and recall scores of 94.94% and 95.89% indicate that the model excels at correctly identifying true positives, showcasing its strong positive prediction accuracy.

**Figure 9:**
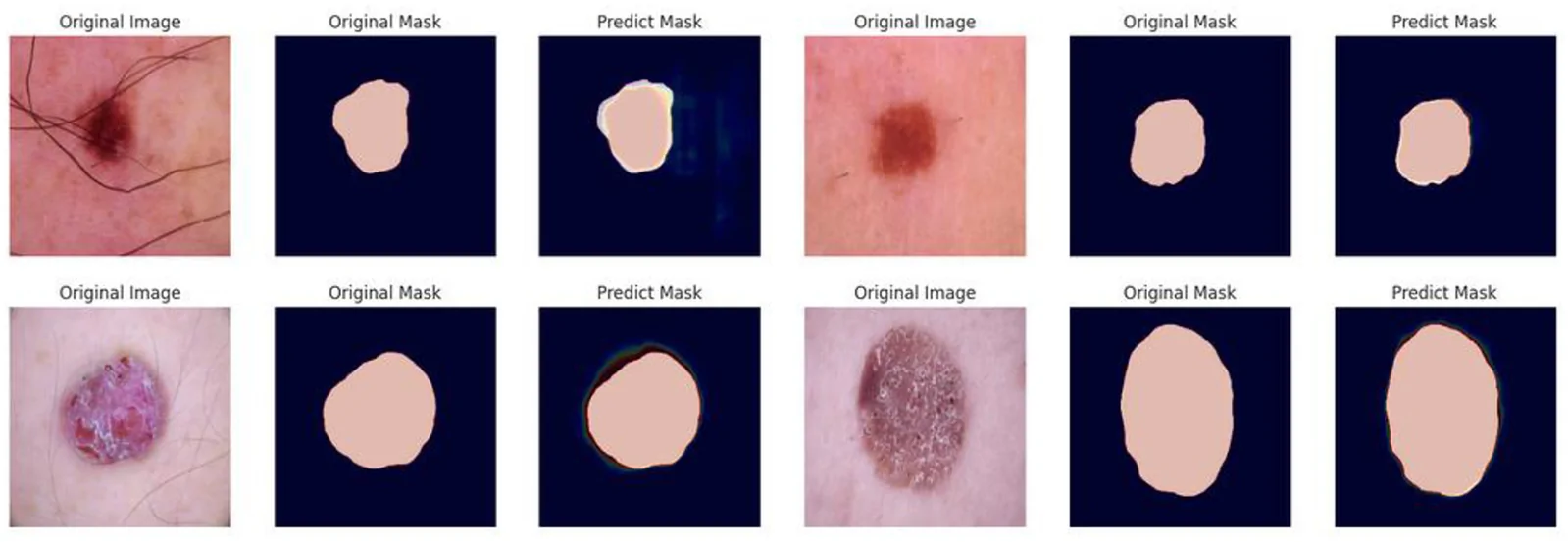
Model performance on unseen data in segmentation.

The consistent performance across different datasets shows the robustness and reliability of the proposed model. Figure 9 illustrates the detection results achieved with the proposed hybrid model across different images. It clearly illustrates the precision of model in segmenting skin lesions accurately.

### 4.3 Ablation study

Ablation studies were performed to examine the individual effects of the lightweight CNN encoder and Vision Transformer module, by turning them off and on gradually. The evaluation shows that every component has an impact on the segmentation performance. The lightweight CNN is able to capture fine-grained local features of the lesion, while the Vision Transformer enhances the global context representation. The proposed hybrid architecture is validated by their combination which provides the best overall segmentation accuracy. The ablation results are summarized in Table 8.

**Table 8:** Ablation study results.

Model Configuration	Accuracy (%)	Dice (%)	Jaccard (%)
CNN Only	92.63	91.74	90.81
ViT Only	93.48	92.91	92.08
CNN + ViT (Proposed)	95.89	94.29	96.91

### 4.4 Computational complexity analysis

We examined the computational efficiency of the proposed hybrid CNN–ViT architecture by analyzing the number of trainable model parameters, floating-point operations (FLOPs), training time, and inference latency. The proposed framework has an efficient computation profile, which comes from the lightweight CNN encoder and the selective integration of the Vision Transformer module. The computational analysis has been carried out in the same experimental environment given in Section 3.1. The results are reported in Table 9.

**Table 9:** Computational complexity analysis of proposed model.

Model	Parameters (M)	FLOPs (G)	Training time	Inference time
Proposed CNN–ViT	8.7M	5.4G	2.6 h	18.7 ms/image

### 4.5 Statistical significance analysis

For statistical reliability of the CNN–ViT framework improvements, multiple independent training runs were performed and the results were analyzed statistically. The proposed model was trained five times using different random seeds and Dice and Jaccard score mean and standard deviations were computed. Moreover, a paired statistical test was performed between the maximum baseline method (Attention Swin U-Net) and the proposed CNN–ViT model to check whether this improvement in the performance was statistically significant. The p-values obtained matched the standard for significance at 0.05, meaning that the improvement that was obtained by the proposed framework was statistically significant, which is not due to random variations. Table 10 presents the mean, standard deviation, and p-value results of the statistical significance analysis.

**Table 10:** Statistical significance analysis of the proposed CNN–ViT model.

Method	Dice (%) Mean ±Std	Jaccard (%) Mean ±Std	*p*-value
Attention Swin U-Net	93.70 ± 0.42	88.20 ± 0.51	—
Proposed CNN–ViT	**94.59** **±0.28**	**94.43** **±0.31**	**< 0.05**

Bold values indicate the best performance among the compared methods.

### 4.6 Clinical deployment challenges and future directions

The proposed CNN–ViT framework achieves promising segmentation performance but faces several challenges to the time of clinical deployment. Variations in imaging devices, acquisition conditions, and patient populations could impact model generalization. Thus, multi-center validation, workflow integration and regulatory aspects are required to ensure clinical reliability. Moreover, the lack of interpretability of deep learning models is still a significant issue in healthcare. Future studies aim to combine Explainable AI (XAI) methodologies including attention visualization, Grad-CAM and feature attribution techniques in the aim of pinpointing salient regions of the image and increasing the level of trust from the clinicians. Further research will focus on lightweight transformer design, privacy-preserving learning, and clinical testing in the real world to further improve the scalability and deployment of the transformer.

## 5 Conclusion

This study focuses on the challenge of accurately and efficiently segmenting skin lesions from dermoscopic images. We present a novel Hybrid CNN-ViT segmentation model tailored for skin images. The novel model used a lightweight convolutional module for feature extraction with ViT module to capture global attention for better representation learning. This hybrid model enhances the accuracy of lesion segmentation. After the data preparation, we explored various factors in the training phase of the model, including the number of training epochs, batch size, and choice of optimizers, to boost model performance. After the training of the model on the ISIC 2020 dataset, we used two datasets for the generalizability of the model. These datasets include PH2 and Ham10000. Our model achieved an impressive accuracy of 95.89% on the ISIC 2020 dataset using the SGD optimizer in the training phase. The proposed model also achieved handsome results in the generalization phase, with 94.61% and 95.68% accuracy on PH2 and HAM10000, respectively. These findings represent significant strides forward in the field of skin lesion segmentation, which could play a crucial role in early diagnosis and improving treatment strategies. Although the results look promising, we still need more research to make the model stronger and applicable in different situations. By looking into approaches such as transfer learning and ensemble techniques and by fine-tuning the Vision Transformer layers. We can also work on hybrid transformers to get more reliable results in future.

## Data Availability

The original contributions presented in the study are included in the article/supplementary material, further inquiries can be directed to the corresponding authors.
